# Nucleated red blood cells in the blood of medical intensive care patients indicate increased mortality risk: a prospective cohort study

**DOI:** 10.1186/cc5932

**Published:** 2007-06-05

**Authors:** Axel Stachon, Elmar Segbers, Tim Holland-Letz, Reiner Kempf, Steffen Hering, Michael Krieg

**Affiliations:** 1 Institute of Clinical Chemistry, Transfusion and Laboratory Medicine, BG-University Hospital Bergmannsheil, Buerkle de la Camp-Platz 1, Ruhr-University Bochum, 44789 Bochum, Germany; 2 Department of Medical Informatics, Biometry, and Epidemiology, Overbergstrasse 17, Ruhr-University Bochum, 44801 Bochum, Germany; 3 Department of Internal Medicine, BG-University Hospital Bergmannsheil, Buerkle de la Camp-Platz 1, Ruhr-University Bochum, 44789 Bochum, Germany

## Abstract

**Introduction:**

In critically ill patients, the appearance of nucleated red blood cells (NRBCs) in blood is associated with a variety of severe diseases. Generally, when NRBCs are detected in the patients' blood, the prognosis is poor.

**Methods:**

In a prospective study, the detection of NRBCs was used for a daily monitoring of 383 medical intensive care patients.

**Results:**

The incidence of NRBCs in medical intensive care patients was 17.5% (67/383). The mortality of NRBC-positive patients was 50.7% (34/67); this was significantly higher (*p *< 0.001) than the mortality of NRBC-negative patients (9.8%, 31/316). Mortality increased with increasing NRBC concentration. Seventy-eight point six percent of the patients with NRBCs of more than 200/μl died. The detection of NRBCs is highly predictive of death, the odds ratio after adjustment for other laboratory and clinical prognostic indicators being 1.987 (*p *< 0.01) for each increase in the NRBC category (0/μl, 1 to 100/μl, 101 to 200/μl, and more than 200/μl). Each step-up in the NRBC category increased the mortality risk as much as an increase in APACHE II (Acute Physiology and Chronic Health Evaluation II) score of approximately 4 points. The mortality of patients who were NRBC-positive on the day of relocation from the intensive care unit to a peripheral ward was 27.6% (8/27). This was significantly higher than the mortality of patients who were NRBC-negative on the relocation day (8.6%, 28/325; *p *< 0.01). On average, NRBCs were detected for the first time 14 days (median, 3 days) before death.

**Conclusion:**

The routine analysis of NRBCs in blood is of high prognostic power with regard to mortality of critically ill patients. Therefore, this parameter may serve as a daily indicator of patients at high mortality risk. Furthermore, NRBC-positive intensive care patients should not be relocated to a normal ward but should obtain ongoing intensive care treatment.

## Introduction

Under normal conditions, the peripheral blood of healthy adults is generally free of nucleated red blood cells (NRBCs), which tend to be found in patients with severe diseases [1-5] who have a relatively poor prognosis [3,4,6-9]. In most of the earlier studies on NRBCs, the concentration was determined microscopically by a stained peripheral blood smear. With such a technique, it is difficult to detect NRBC concentrations of less than 200/μl [10]. For several years, a more convenient and sensitive technique has been available in the form of mechanized blood analyzers. With such an analyzer, one can routinely determine NRBC concentrations of less than 100/μl [11-13] The results of our recent studies with this new technique indicate that the detection of NRBCs may serve as an early indicator in patients at increased risk of mortality: on average, the presence of NRBCs was detected 1 to 3 weeks before death [14,15]. Furthermore, the analysis of the cytokine profile in the blood of NRBC-positive patients (without hematologic diseases) suggests that NRBCs may be considered a parameter that sums hypoxic and inflammatory injuries. This may be the reason why the appearance of NRBCs is a strong predictor of increased mortality [15-17].

Recently, we reported on the poor prognosis of surgical intensive care patients when NRBCs are found in the peripheral blood [18]. In that study, for the first time, a systematic day-to-day screening for NRBCs in the blood of surgical intensive care patients was performed. Our study revealed that 32% of the surgical intensive care patients were NRBC-positive at least once. The detection of NRBCs was associated with a greatly increased mortality of 44% (versus 4% of NRBC-negative patients). The area under the curve amounted to 0.86. On average, NRBCs were detected nine days before death. Therefore, in the present study, we set out to establish whether the daily screening for NRBCs in medical intensive care patients could serve as an early indicator of medical intensive care patients at extremely high risk.

Our study revealed that 18% of the medical intensive care patients were NRBC-positive. The detection of NRBCs was associated with a greatly increased in-hospital mortality. More than 50% of the NRBC-positive patients died. Furthermore, the mortality was three times higher in patients who were NRBC-positive on the day of relocation from the intensive care unit to a peripheral ward compared to patients who were NRBC-negative on the relocation day. On average, NRBCs were detected 14 days before death. These results suggest that the routine daily measurement of NRBCs could aid in a daily risk assessment of medical intensive care patients.

## Materials and methods

### Subjects and protocol

All intensive care patients treated between April 2003 and January 2004 in the intensive care unit of the Department of Internal Medicine of Berufsgenossenschaftliche Universitaetsklinik Bergmannsheil GmbH (University Hospital, Ruhr-University Bochum, Germany) (*n *= 383) were included in this study. Patients younger than 18 years and patients after surgery were excluded from this study. To evaluate the prognostic significance of NRBCs in the peripheral blood of medical intensive care patients, we screened one blood sample of each patient each day by means of a Sysmex XE-2100 (Sysmex Europe GmbH, Norderstedt, Germany). Blood samples were routinely drawn in the morning. For statistical analysis, a patient was defined as NRBC-positive when NRBCs were detected in the blood at least once. Outcome was considered as in-hospital mortality. Ethical approval to conduct this study was granted by the Ethical Committee of Ruhr-University Bochum (reference no. 1982).

### Laboratory tests

Blood count parameters (NRBCs, leukocytes, hemoglobin, and thrombocytes) were measured using a Sysmex XE-2100 blood analyzer in line with the manufacturer's recommendations. According to the manufacturer, the NRBC detection limit was greater than 19/μl. Stringent internal quality control measurements were performed, and the criteria of acceptance were fulfilled throughout.

Creatinine, alanine aminotransferase, and C-reactive protein were measured with an LX 20 analyzer (Beckman Coulter GmbH, Krefeld, Germany), and prothrombine time ratio was assayed with a BCS (Behring Coagulation System) (Dade Behring, Schwalbach, Germany), all in accordance with the recommendations of the manufacturers. The quality assurance of quantitative determinations was strictly performed according to the German Norm: Quality Assurance in Medical Laboratories (DIN [Deutsches Institut für Normung] 58936, 2000). The criteria of acceptance were fulfilled throughout. Retrospective analysis of the laboratory data revealed 0.3% missing values.

### Statistical analysis

Data are presented as the mean ± standard error of mean. When samples were normally distributed, the differences between the data for survivors and deceased were analyzed using the *t *test procedure. When samples were not normally distributed, the Mann-Whitney test was used because this test does not require a normal distribution of data. In the case of categorical data, the Fisher exact test was used. Correlations were analyzed by Pearson or non-parametric Spearman correlation. A *p *value of less than 0.05 was considered statistically significant.

A receiver operating characteristic curve was obtained by plotting the true-positive proportion (sensitivity) against the false-negative proportion (1 – specificity). The area under the curve (C-statistics) was calculated by non-linear regression.

The prognostic significance of NRBCs and other risk indicators was assessed using multiple logistic regression. In this study, the logistic regression tries to estimate the relative effect that parameters have on the patients' outcome. This is facilitated by assuming a functional relationship (the 'logistic model') between variables and probability of outcome. Then, for all possible settings, every variable is given a relative importance that makes the actual observed event 'most likely', taking into account the effects of all other variables. This is called the 'maximum likelihood' estimate of the variables' influence. These coefficients provide a relative weighting for each variable. Moreover, they can be used to derive odds ratios for the variables. If the odds ratio differs significantly from 1, a significant prognostic power that is independent of the other variables considered may be assumed.

In a first step, laboratory data were analyzed with regard to mortality. If reasonable for calculation of the odds ratios, the data were categorized in up to four categories. That is, NRBCs were subdivided into four categories: 0/μl, 1 to 100/μl, 101 to 200/μl, and more than 200/μl. A backward selection multiple logistic regression analysis was performed by first including all parameters in a multivariate model and subsequently leaving out the parameters with the largest *p *values until no parameter with a *p *value greater than 0.25 was included. The calculations were carried out using SAS version 8.02 (SAS Institute Inc., Cary, NC, USA). The intention of this study was to evaluate the prognostic power of the presence of NRBCs in the blood with regard to the patients' in-hospital mortality risk.

### Nucleated red blood cells and established risk models for intensive care patients

The prognostic significance of NRBCs was evaluated under consideration of established risk models: the Acute Physiology and Chronic Health Evaluation II (APACHE II) (first evaluated in 1985 [19]) and the Simplified Acute Physiology Score II (SAPS II) (first evaluated in 1993 [20]). The APACHE II severity index includes the following risk factors: body temperature, mean arterial pressure, heart rate, respiratory rate, blood oxygenation, arterial pH, sodium, potassium, creatinine, hematocrit, white blood cell count, Glasgow coma scale, age, and anamnestic data concerning severe organ insufficiency or immunocompromised states of health. The SAPS II considers the following risk factors: age, heart rate, systolic blood pressure, body temperature, blood oxygenation, urinary output, urea, white blood cell count, potassium, sodium, bicarbonate, bilirubin, Glasgow coma scale, chronic diseases (that is, malignancies and acquired immunodeficiency syndrome), and type of admission (that is, medical and unscheduled surgical). Both the APACHE II score and the SAPS II are determined from the most deranged (worst) physiologic value (for example, the lowest blood pressure or the highest white blood cell count) during the initial 24 hours after intensive care unit admission.

## Results

### Patient characteristics

We included 383 medical intensive care patients. The mean age was 66.3 ± 0.8 years (range, 20 to 94 years). Two hundred twenty-five male (58.7%) and 158 female (41.3%) patients were included in this study. On average, patients were treated for 4.1 ± 0.3 days (*n *= 383) in the intensive care unit. Total mortality was 17.0% (65/383). The APACHE II score and the SAPS II amounted to 16.0 ± 0.5 and 35.2 ± 0.9, respectively.

**Figure 1:**
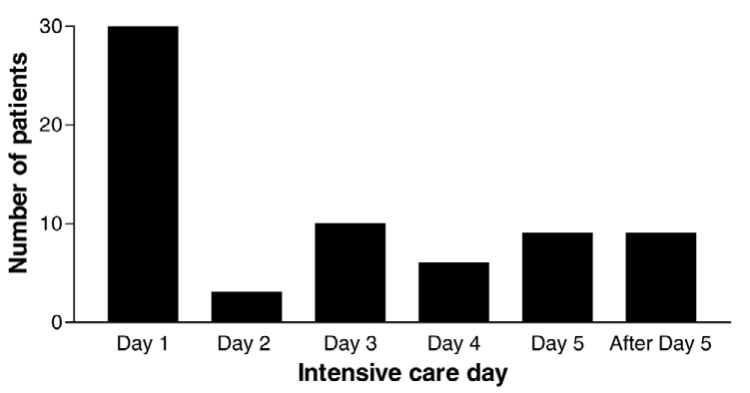
Intensive care days on which nucleated red blood cells were detected for the first time in the blood of medical intensive care patients.

The incidence of NRBCs in the blood was 17.5% (67/383). No significant difference was found between the incidences in male (16.4%, 37/225) and female (19.0%, 30/158) patients. On the day of admission, 7.8% (30/383) of the patients were NRBC-positive. On average, NRBCs were detected for the first time on the third day of intensive care treatment (3.1 ± 0.4; Figure 1), but in 44.8% (30/67) of the NRBC-positive patients, NRBCs were already detected on the admission day. On average, the highest NRBC concentration of each individual NRBC-positive patient was 189 ± 41/μl (range, 20 to 1,760/μl; median, 80/μl; *n *= 67). Some of the basal clinical characteristics of NRBC-positive and NRBC-negative patients are summarized in Table 1.

**Table 1:** Clinical data and main diagnosis of treatment of NRBC-positive (*n *= 67) and NRBC-negative (*n *= 316) patients

Parameter	NRBC-positive	NRBC-negative	*P*
Age, years	68 ± 2	66 ± 1	n.s.
Gender, male/female	55%/45%	59%/41%	n.s.
Intensive care treatment, days	8.7 ± 1.2	3.2 ± 0.2	< 0.001
Body mass index	27.8 ± 0.9	26.5 ± 0.3	n.s.
Mortality	50.7%	9.8%	< 0.001
APACHE II score points	21.5	14.9	< 0.001
SAPS II points	47.5	32.6	< 0.001
Acute coronary syndrome/AMI	16.4% (*n *= 11)	38.0% (*n *= 120)	< 0.001
Cardiac arrhythmia	10.4% (*n *= 7)	14.2% (*n *= 45)	n.s.
Cardiac insufficiency	11.9% (*n *= 8)	6.6% (*n *= 21)	n.s.
Pulmonary diseases	23.9% (*n *= 16)	8.9% (*n *= 28)	< 0.01
Gastrointestinal diseases	16.4% (*n *= 11)	7.9% (*n *= 25)	< 0.05
Cerebral diseases	6.0% (*n *= 4)	10.1% (*n *= 32)	n.s.
Toxication	4.5% (*n *= 3)	4.1% (*n *= 13)	n.s.
Metabolic diseases	1.5% (*n *= 1)	4.4% (*n *= 14)	n.s.
Other diagnosis	9.0% (*n *= 6)	5.7% (*n *= 18)	n.s.

AMI, acute myocardial infarction; APACHE II, Acute Physiology and Chronic Health Evaluation II; NRBC, nucleated red blood cell; n.s., not significant; SAPS II, Simplified Acute Physiology Score II.

### Prognostic significance of nucleated red blood cells

**Figure 2:**
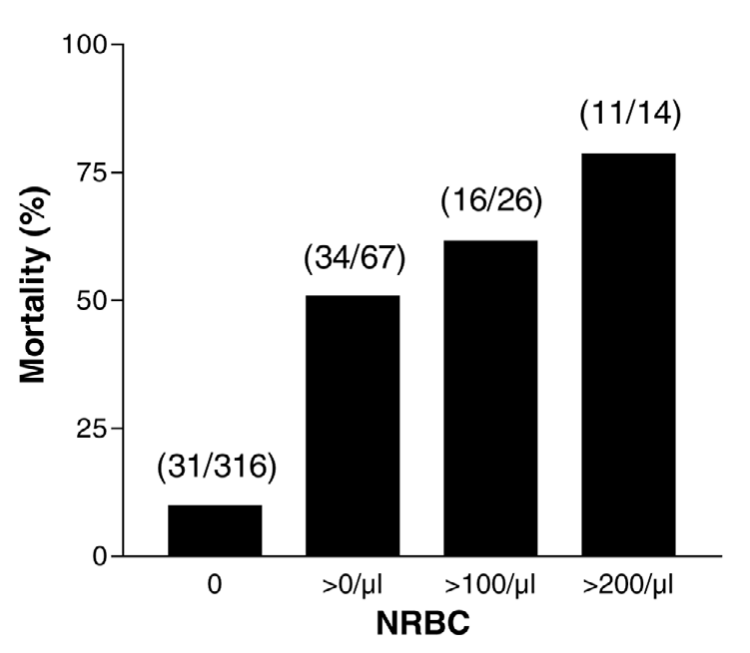
In-hospital mortality of medical intensive care patients in relation to the concentration of nucleated red blood cells (NRBCs) in the blood. Numbers in parenthesis denote the ratio of deceased patients to all patients with the respective NRBC concentration.

The mortality of NRBC-positive patients was 50.7% (34/67). The predictive value for death increased with higher NRBC concentrations (Figure 2). The mortality was 46.7% (14/30) in patients who were NRBC-positive on the day of admission to the intensive care unit. In contrast, the mortality of NRBC-negative patients was 9.8% (31/316; *p *< 0.001).

Furthermore, the mortality of patients who were NRBC-positive on the day of relocation from the intensive care unit to a peripheral ward was 27.6% (8/27). This was significantly higher than the mortality of patients who were NRBC-negative on the relocation day (8.6%, 28/325; *p *< 0.01).

Overall, with regard to in-hospital mortality, NRBCs in blood showed sensitivity and specificity of 52.3% and 89.6%, respectively. The area under the curve was 0.72.

NRBCs were an early indicator of patients at increased mortality risk. On average, in NRBC-positive patients who died, NRBCs were detected for the first time 13.6 ± 3.8 days (median, 3 days; *n *= 34) before death.

After the first detection of NRBCs in blood and during the further course of intensive care treatment, when the NRBCs have disappeared from the circulation, the mortality again decreased. That is, when former NRBC-positive patients were again NRBC-negative for more than 4 days after the final detection of NRBCs in blood, the mortality decreased to 16.7% (1/6).

**Figure 3:**
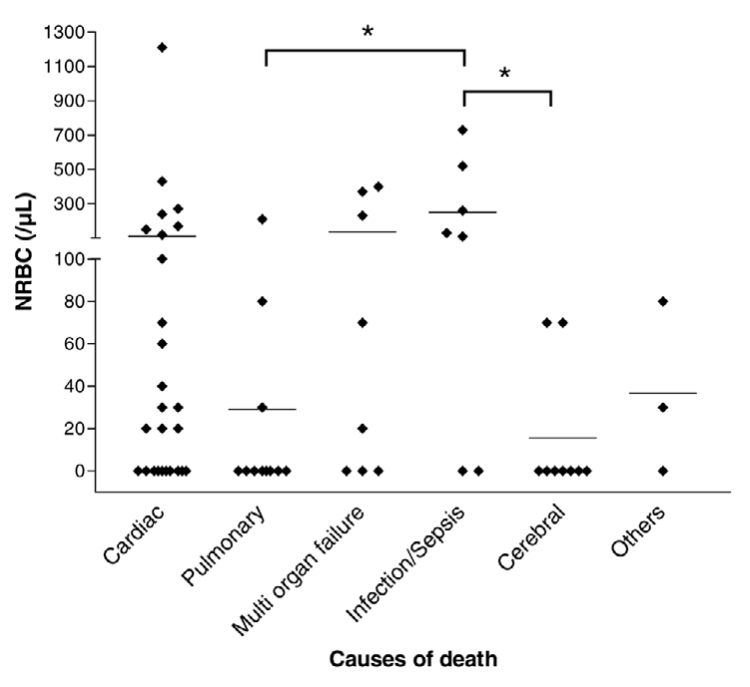
Concentration of nucleated red blood cells (NRBCs) in the blood of medical intensive care patients who have died from various causes. ◆ indicate the NRBC concentration of each individual deceased patient. The average concentration is indicated by horizontal bars.  denote the significance of the difference.

As shown in Figure 3, the appearance of NRBCs in blood seems not to be associated with one particular cause of death. However, patients who have died from infections or sepsis, in particular, had significantly higher NRBC concentrations than patients who have died from cerebral or pulmonary complications. None of the other defined causes of death was associated with an NRBC concentration that was significantly higher than the others.

### Nucleated red blood cells in relation to other clinical and laboratory risk indicators

The incidence of NRBCs increased with higher APACHE II and SAPS II scores (Table 2). Accordingly, the Spearman correlation revealed a significant correlation between the NRBC concentration and the APACHE II (*r *= 0.292, *p *< 0.001) and the SAPS II scores (*r *= 0.320, *p *< 0.001).

**Table 2:** Incidence of NRBCs in blood in medical intensive care patients in relation to the APACHE II and the SAPS II

Risk model	Score range of risk model	Incidence of NRBCs
APACHE II	< 11	4.7% (6/127)
	11–20	18.4% (28/152)
	21–30	30.6% (22/72)
	> 30	34.4% (11/32)
		
SAPS II	< 21	5.6% (4/71)
	21–40	13.3% (27/203)
	41–60	34.3% (23/67)
	> 60	30.9% (13/42)

APACHE II, Acute Physiology and Chronic Health Evaluation II; NRBC, nucleated red blood cell; SAPS II, Simplified Acute Physiology Score II.

The Spearman correlation of the NRBCs with other laboratory parameters is displayed in Table 3. When correlation was calculated with values measured on the day of the first appearance of NRBCs in blood, NRBCs significantly increased with the leukocytes (*r *= 0.373, *p *< 0.01) and the creatinine concentration (*r *= 0.284, *p *< 0.05). Moreover, NRBCs increased with a decreasing prothrombin time ratio (*r *= -0.408, *p *< 0.001). The concentrations of hemoglobin, thrombocytes, and C-reactive protein as well as the alanine aminotransferase activity were not significantly correlated with the NRBC concentration.

**Table 3:** Spearman correlation of the nucleated red blood cell concentration with other laboratory parameters (*n *= 67)

Parameter	*r*	*P*
Hemoglobin	-0.113	n.s.
Leucocytes	0.373	< 0.01
Thrombocytes	-0.152	n.s.
Creatinine	0.284	< 0.05
Prothrombin time ratio	-0.408	< 0.001
Alanine aminotransferase	0.172	n.s.
C-reactive protein	0.169	n.s.

Correlation was calculated with values measured on the day of the first appearance of nucleated red blood cells in blood. n.s., not significant.

The detection of NRBCs was an independent risk indicator of poor outcome. In terms of mortality, the odds ratio for each stepwise increase in the NRBC categories was calculated in relation to other clinical and laboratory risk indicators by means of multiple logistic regression (Table 4). Because the correlation coefficient calculated by linear regression between APACHE II score and SAPS II was *r *= 0.91, only the APACHE II score was considered for the multiple logistic regression. The detection of NRBCs is highly predictive of death, the odds ratio after adjustment for other clinical and laboratory prognostic indicators being 1.987 (*p *< 0.01) for each increase in the NRBC category (0/μl, 1 to 100/μl, 101 to 200/μl, and more than 200/μl). That is, patients with more than 200/μl NRBCs had a more than seven-fold higher risk of dying than patients without NRBCs.

**Table 4:** Multivariate odds ratio estimates of clinical and laboratory risk indicators for in-hospital mortality calculated by logistic regression (*n *= 383)

Parameter	Point estimate	95% confidence limits	*P*
NRBCs^a^	1.987	1.211–3.261	< 0.01
Leukocytes (> 10/nl)	0.480	0.178–1.294	0.147
Prothrombin time ratio (< 60%)	3.968	1.733–9.090	< 0.01
Alanine aminotransferase^b^	1.223	0.932–1.620	0.162
C-reactive protein^c^	1.214	0.901–1.635	0.202
APACHE II	1.168	1.112–1.227	< 0.001

Age, gender, body mass index, APACHE II score, the highest nucleated red blood cell (NRBC) concentration, the highest creatinine concentration, the lowest hemoglobin concentration, the lowest thrombocytes concentration, the highest leukocyte concentration, the highest alanine aminotransferase activity, the highest C-reactive protein concentration, and the lowest prothrombin time ratio were considered for the calculation. All risk indicators with *p *values greater than 0.25 were removed from the model. ^a^Subdivided into four categories (0/μl, 1 to 100/μl, 101 to 200/μl, and more than 200/μl); odds ratio was calculated for each stepwise increase in the category. ^b^After log transformation. ^c^Subdivided into four categories (0 to 5.0 mg/dl, 5.1 to 10.0 mg/dl, 10.1 to 15.0 mg/dl, and more than 15 mg/dl); odds ratio was calculated for each stepwise increase in the category. APACHE II, Acute Physiology and Chronic Health Evaluation II.

Furthermore, under consideration of the APACHE II score, the 'maximum likelihood estimate' for each increase in the NRBC category was 0.687 ± 0.253 and therefore approximately four times (exactly 4.40 times) higher than the 'maximum likelihood estimate' for each one-point increase in the APACHE II score (0.156 ± 0.025). Therefore, each step-up in the NRBC category is equivalent to approximately 4 APACHE II score points. Consequently, an adjustment of the APACHE II score could be performed by adding those 4, 8, or 12 points dependent on the patient's NRBC category (1 to 100, 101 to 200, and more than 200/μl) to the individual APACHE II score of NRBC-positive patients. In practice, this modification would be a reassessment of the patient's prognosis. The area under the curve for this modified APACHE II score was 0.91 compared to 0.87 and 0.72 for the APACHE II score and the NRBCs alone, respectively (Table 5).

**Table 5:** C-statistics for several risk indicators for in-hospital mortality of medical intensive care patients

Parameter	Area under curve
NRBC, highest value	0.72
Leukocytes, highest value	0.61
Prothrombin time ratio, lowest value	0.73
Alanine aminotransferase, highest value	0.73
C-reactive protein, highest value	0.72
SAPS II, on admission	0.88
APACHE II, on admission	0.87
APACHE II, on admission + NRBC, highest value^a^	0.91

Only data from the intensive care unit were considered. ^a^The APACHE II score was incremented under consideration of the NRBC concentration as follows: NRBC 0/μl: +0, NRBCs 1 to 100/μl: +4, NRBCs 101 to 200/μl: +8, NRBCs more than 200/μl: +12). APACHE II, Acute Physiology and Chronic Health Evaluation II; NRBC, nucleated red blood cell; SAPS II, Simplified Acute Physiology Score II.

## Discussion

To our knowledge, this is the first study in which the detection of NRBCs in the peripheral blood was investigated with regard to its prognostic significance for the intensive care mortality of medical intensive care patients. In earlier studies, we and others have shown that the detection of NRBCs is associated with a relatively poor prognosis [3,4,6-8,10,14,21,22]. In most of those studies, the NRBC detection and quantification were based on the microscopic analysis of stained blood smears. This technique is time-consuming and only partly suitable for the detection and quantification of NRBC concentrations of less than 200/μl [10].

In this study, the NRBC concentration was screened with a mechanized blood analyzer of high sensitivity [11,12,23]. The present study revealed that approximately 18% of all medical intensive care patients were NRBC-positive at least once. Interestingly, in nearly half of the NRBC-positive patients, NRBCs were detected already on the admission day.

Our data confirmed the high prognostic power of the mechanized detection of NRBCs in blood in terms of mortality. The total in-hospital mortality of NRBC-positive patients of this study was 50.7%. Furthermore, as shown in earlier studies, the present data showed that the mortality increased with an increasing NRBC concentration [10,24,25] Approximately 80% of the patients with NRBC concentrations higher than 200/μl died.

Our study revealed that the daily screening for NRBCs can be used to estimate the patients' mortality risk. Not only did the predictive value for death increase with the concentration of NRBCs in the blood, but the prognosis improved when the NRBC concentration decreased. In particular, after the first detection of NRBCs in blood and during the further course of intensive care treatment, when the NRBCs have disappeared from the circulation for more than four days, the mortality again significantly decreased nearly to values of NRBC-negative patients [18].

In the present study, increased creatinine and leukocyte concentrations and a lower prothrombin time ratio were significantly correlated with increased NRBC concentrations. Although these findings suggest that NRBC-positive patients are more severely burdened than NRBC-negative patients, the detection of NRBCs is an independent predictor of poor outcome. To evaluate the independent attributable risk factor, a logistic regression considering NRBCs, age, gender, body mass index, APACHE II score, creatinine, hemoglobin, thrombocytes, leukocyte, alanine aminotransferase, C-reactive protein, and the prothrombin time ratio was performed. As a result, the independent prognostic power of NRBCs is underlined by an odds ratio of 1.987 for each stepwise increase in the NRBC category. That is, patients with NRBCs of more than 200/μl have a more than seven-fold higher risk to die than NRBC-negative patients. In recent studies, we have already demonstrated that the detection of NRBCs is a risk indicator that is independent of several other established risk indicators [10,14,16,24].

Among the general severity of illness scoring systems for intensive care patients, APACHE II and SAPS II have become two of the most accepted and used [26-30]. However, the present data suggest that the APACHE II score could be significantly improved by adding up to 12 score points, considering the presence of NRBCs as an independent variable in this score, as suggested for the abbreviated burn severity index in patients with burns [25].

The analysis of the lifespan of the patients who died indicates that NRBCs in blood were found not just immediately before death. Moreover, our present study showed that the detection of NRBCs is often a relatively early phenomenon prior to death. In deceased patients, NRBCs were detected 14 days before death. Therefore, NRBCs would seem to be an early indicator of increased risk.

Finally, the underlying pathophysiology of NRBCs in blood is not fully understood. In our study, no association with only one of the various causes of patient death was found. However, some authors have claimed that hypoxemia [31,32], acute and chronic anemia [33,34], or severe infections [35,36] are linked to the appearance of NRBCs in critically ill patients. In this context, we recently reported on the cytokine profile and the erythropoietin concentrations in NRBC-positive patients [17]. Our data suggested an important role of inflammation and/or decreased tissue oxygenation (caused by local or systemic circulatory disorders) for the appearance of NRBCs in blood. NRBCs may thus be considered a marker that sums up hypoxic and inflammatory injuries. It seems obvious that these complications have an impact on patient prognosis. Therefore, this could be the reason why the appearance of NRBCs is a strong predictor of increased mortality.

However, concerning such an association between inflammation (with or without hypoxia) and NRBCs, it is attractive to speculate what kind of therapy could improve the poor prognosis of NRBC-positive patients. Currently, studies are under way in our university hospital to show whether intensifying the treatment of patients with NRBCs (that is, an earlier administration of antibiotics or an anti-inflammatory therapy) can reduce their mortality rate.

Nonetheless, we observed that the mortality was three times higher in patients who were NRBC-positive on the day of relocation from the intensive care unit to a normal ward compared to patients who were NRBC-negative on the relocation day. Consequently, it seems obvious that NRBC-positive patients should obtain ongoing intensive care treatment.

## Conclusion

This is the first study in which the daily screening for NRBCs in the peripheral blood of patients in the medical intensive care unit was investigated with regard to its prognostic power for in-hospital mortality. The incidence of NRBC-positive patients was 18%. NRBC detection in critically ill patients was associated with significantly increased in-hospital mortality (50.7% versus 9.8%). The predictive value for death increased with the NRBC concentration and seems to decline again when the NRBCs have disappeared from the circulation. The prognostic significance of NRBCs was independent of other laboratory and clinical risk parameters. An improvement of established risk models like APACHE II seems feasible. Furthermore, the detection of NRBCs in blood is a relatively early phenomenon prior to death, so screening for NRBCs may aid in the early identification of patients at high risk. Further studies are needed to clarify whether the detection of NRBCs could help to decide on a change of patient management, but our present data suggest that NRBC-positive patients should obtain ongoing intensive care treatment.

## Key messages

• The detection of NRBCs in the blood of medical intensive care patients is associated with significantly increased in-hospital mortality (50.7% versus 9.8%). The prognostic significance of NRBCs was independent of other laboratory and clinical risk parameters. The predictive value for death increased with the NRBC concentration and seems to decline again when the NRBCs have disappeared from the circulation.

• Our present data suggest that NRBC-positive patients should obtain ongoing intensive care treatment.

## Abbreviations

APACHE II = Acute Physiology and Chronic Health Evaluation II; NRBC = nucleated red blood cell; SAPS II = Simplified Acute Physiology Score II.

## Competing interests

AS and RK obtained a grant from Sysmex Europe GmbH (Norderstedt, Germany) to perform this study. The other authors declare that they have no competing interests.

## Authors' contributions

All authors made substantive intellectual contributions to the design and conception of this study. AS was responsible for data acquisition and data presentation, performed the analysis and interpretation of data, and was responsible for the writing of the manuscript. RK and ES were responsible for data acquisition and data presentation and performed the analysis and interpretation of data. SH was responsible for data acquisition and data presentation. TH-L performed the analysis and interpretation of data. MK was responsible for the writing of the manuscript. All authors read and approved the final manuscript.
